# Structural and functional changes in the retina in Parkinson’s disease

**DOI:** 10.1136/jnnp-2022-329342

**Published:** 2023-02-17

**Authors:** Jordan Nicolas Alves, Britta U. Westner, Andreas Højlund, Rimona Sharon Weil, Sarang S. Dalal

**Affiliations:** 1 Center of Functionally Integrative Neuroscience, Aarhus University, Aarhus, Denmark; 2 Radboud University, Donders Institute for Brain, Cognition and Behaviour, Nijmegen, the Netherlands; 3 Department of Linguistics, Cognitive Science & Semiotics, Aarhus University, Aarhus, Denmark; 4 Dementia Research Centre, University College London, 8-11 Queen Square, London, WC1N 3AR, UK; 5 Wellcome Centre for Human Neuroimaging, University College London, 12 Queen Square, London, WC1N 3AR, UK; 6 Movement Disorders Centre, University College London, London WC1N 3BG, UK

**Keywords:** Parkinson’s disease, electroretinography, optical coherence tomography, dopamine, retina

## Abstract

Parkinson’s disease is caused by degeneration of dopaminergic neurons, originating in the *substantia nigra pars compacta*, and characterized by bradykinesia, rest tremor and rigidity. In addition, visual disorders and retinal abnormalities are often present and can be identified by decreased visual acuity, abnormal spatial contrast sensitivity or even difficulty in complex visual task completion. Because of their early onset in de novo Parkinson’s disease patients, the anatomical retinal changes and electrophysiological modification could be valuable markers even at early stages of the disease. However, due to the concomitant occurrence of normal aging, the relevance and specificity of these predictive values can be difficult to interpret. This review examines retinal dysfunctions arising in Parkinson’s disease. We highlight the electrophysiological delays and decreased amplitude in the electroretinography recorded in patients and animal models. We relate this to coexisting anatomical changes such as retinal nerve fiber layer and macular thinning, measured using optical coherence tomography, and show that functional measures are more consistent overall than optical coherence-measured structural changes. We review the underlying chemical changes seen with loss of retinal dopaminergic neurons, and the effect of levodopa treatment on the retina in Parkinson’s disease. Finally, we consider whether retinal abnormalities in Parkinson’s disease could have a role as potential markers of poorer outcomes and help stratify patients at early stages of the disease. We emphasize that retinal measures can be valuable, accessible, and cost-effective methods in the early evaluation of Parkinson’s disease pathogenesis with potential for patient stratification.

## Introduction

**Figure 1:**
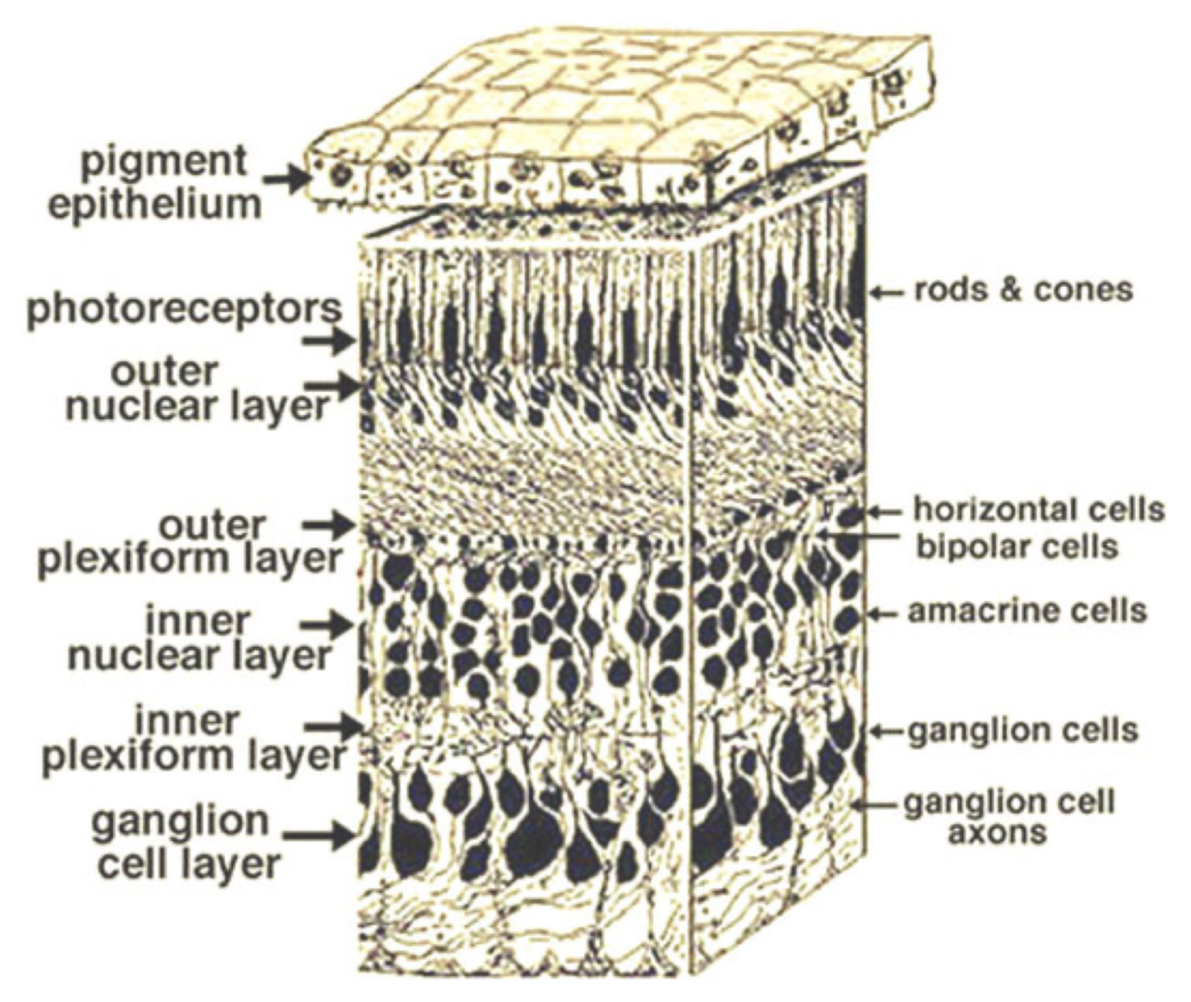
Portion of a human retina from Helga et al.^9^ Copyrights information: Attribution-Noncommercial 4.0 International Creative Commons license (CC BY-NC 4.0).

Research on Parkinson’s disease has broadened over time to consider exteroception deficits (such as touch, pressure, taste, olfaction, and visual deficits) and their link to the quality of life as well as their potential for predicting disease progression. Visual symptoms, in particular, are common with studies reporting that up to 70% of patients with Parkinson’s disease will express recurrent visual complaints.^1^ Ocular symptoms are also described, such as dry eyes^2^ and blepharitis, possibly due to a decreased blink rate. These ocular problems have direct consequences on patients’ abilities to perform daily tasks such as reading, writing, driving or navigating.^3^ Most visual symptoms such as diplopia, difficulty reading, freezing in narrow spaces, and hallucinations are more common in Parkinson’s dementia than in patients with normal cognition^4^ and visual dysfunction in Parkinson’s disease appears to relate to poorer outcomes including depression, dementia and shorter survival.^5,6^ Although many visual symptoms are due to impaired high-level visual processing, earlier stages of visual processing are also affected,^7,8^ and changes in retinal structure and function can also be found in patients with Parkinson’s disease such as color discrimination as well as lower contrast sensitivity and visual acuity. Indeed, in a unified conception of the visual pathway, abnormalities stemming from the retina may become aberrant input to visual cortex, affecting functions such as visuospatial construction, object perception, face and emotion recognition as well as their visual acuity, contrast sensitivity, color vision, and motion perception.^7^ Here, we will review evidence for changes in retinal structure, assessed using optical coherence tomography (OCT) (and see Box 1, Fig.1 for retinal anatomy description), as well as retinal function, as assessed using electroretinography (ERG). See Box 1 for a summary of relevant retinal anatomy. We will also consider retinal changes observed post-mortem, and neurotransmitters likely to be implicated. Finally, we will reflect on the role of retinal dysfunction in stratifying patients to predict future outcomes.

## Methods

Our Search strategy consisted of using relevant databases, combined with specific advanced search engines and keywords in order to screen the available literature. We explored the following databases: (1) PubMed, (2) Scopus, (3) Google Scholar and (4) ScienceDirect. The specific keywords we used were: “Parkinson’s disease”, “Electroretinography”, “Optical coherence tomography”, “Dopamine”, “ERG”, “OCT” and a combination of them. After refinement of our search terms, an example search term for PubMed was: ((Electroretinography)[Title/Abstract] OR (ERG)[Title/Abstract]) AND (Optical coherence tomography)[Title/Abstract] OR (OCT)[Title/Abstract]) AND (Parkinson’s disease) [Title/Abstract]) AND (review[Publication Type]) OR (clinical trial[Publication Type])). From the retrieved articles we selected relevant articles based on eligibility criteria as well as based on the presence of the keywords in the title or the abstract of the article and the following inclusion criteria were: a) Related to our targeted clinical population of Parkinson’s disease; b) Using the relevant techniques: (1) OCT, (2) ERG and (3) Parkinson’s disease assessment scales; c) Investigating the effect of Parkinson’s disease treatment on either: (1) animal models or (2) two groups of participants (healthy matched controls and patients with Parkinson’s disease). Finally, a manual search was conducted through three specific methods: 1) We manually searched the reference list of included articles; 2) We performed a citation tracking where we explored the articles that cite one of the included articles; 3) We followed the “related to” or “similar” articles.

## Optical coherence tomography in Parkinson’s disease patients

Ocular coherence tomography (OCT) employs a super-luminescent diode to illuminate tissues, using either time-domain or spectral domain imaging to assess the eye’s structure, thickness and volume through non-invasive low-coherence reflectometry imaging of internal tissues.^11^ A potential role of OCT during the prodromal or early phase of the disease is to look for early markers of disease activity^12^ which can be easily accessed and routinely implemented in the clinic, and also to understand the potential anatomical origin of the functional changes experienced by patients and to find possible markers that could be used for early detection and monitoring of the disease, as well as evaluation of the efficacy of therapeutic interventions.

Available reports using OCT describe retinal nuclear layer thinning, decreased macular retinal thickness, macular volume and average retinal nerve fiber layer (RNFL) in Parkinson’s disease patients compared to healthy, matched control participants (see Table 1).^13^ Unlu et al.^14^ reported significant thinning of the macular RNFL, retinal ganglion cell (RGC), inner plexiform layer (IPL), inner nuclear layer (INL), outer plexiform layer (OPL), outer nuclear layer (ONL), photoreceptor layer, retinal pigment epithelium, and peripapillary RNFL. The Hoehn and Yahr scale^15^ score, evaluating Parkinson’s disease symptoms severity, was negatively correlated with the macular RNFL volume and ONL thickness. The study revealed thinning of both inner and outer retinal layers, as well as delayed and decreased amplitudes in the multifocal ERG (mfERG) due to profound neurodegeneration in patients with more advanced phases of Parkinson’s disease (measured as longer disease duration and increased clinical severity). In another study, although total macular volume did not differ significantly from healthy controls, several single-layer retinal volumes were significantly reduced, and the OPL was significantly increased.^16^

However, there are notable conflicting findings for the relationship between Parkinson’s disease and retinal structure. Some studies found a significant reduction in RNFL thickness when comparing patients to healthy controls^17–20^ correlated to duration and severity of disease^17,19^ and even helping to stratify Parkinson’s disease patients.^12^ On the other hand, other studies could not confirm such an effect^21–24^ nor any link with disease duration or severity,^20,24,25^ although they did note differences between some patient groups (e.g. hallucinators).^24^ Another parameter such as sector-specific macular thinning was found in the inferior part of the retina^13,20,22,25^ when compared to healthy controls and when differentiating between patients,^18^ but this reduction is not always found23 or else is seen in different retinal sectors.^17,24^ Retinal layer thinning can sometimes be correlated with scores on clinical scales (i.e, Hoehn and Yahr scales, the UPDRS, Mini-Mental State Examination, The Schwab and England Activities of Daily Living scale) or to measured clinical variables,19 but such a correlation is not universally found.^24^ In an effort to link OCT measurements to motor symptoms in Parkinson’s disease, Liu et al.^26^ measured several indicators such as peripapillary RNFL, RGCIPL, central foveal thickness, macular thickness and macular volume, and showed no correlation to motor symptom scales. However, other studies have shown links between global cognitive function scores thickness of the macular RGCIPL, and disease severity and duration.^20,27^ It is notable that parameters such as macular thickness or volume are not always reduced,^13,17^ and some layer specific findings are only sporadically reported such as (1) thinning of the INL and parafoveal region,^24^ the GCIPL^20^ or even the foveal region19; (2) visual acuity and contrast sensitivity reduction^22^ or (3) glaucomatous-like defects.^23^

The inconsistencies between structural retinal measures and Parkinson’s disease clinical scores may relate to factors such as recruitment of patients at different disease stages or differences in OCT hardware, as well as analysis techniques. Despite divergent findings, OCT could still be used to define cutoffs or anatomical benchmarks that could serve clinical purposes. For example, using a multivariate logistic model to predict visual impairment from OCT measurements, the combined value of age and retinal ganglion cell inner plexiform layer (RGCIPL) thickness has high predictive accuracy in distinguishing patients with low visual acuity, poor visual attention, low processing speed and visual perception from healthy participants.^28^ The RGCIPL layer thickness in the parafovea was also the parameter most frequently correlated with visual outcomes in Parkinson’s disease patients.^20,26,27^ Studies combining OCT with retinal functional measures, such as electrophysiology, show more consistent results in Parkinson’s disease. For example, Garcia-Martin et al.^19^ studied the relation between anatomical parameters, electroretinogram (ERG) traces and patients’ overall quality of life and outcome severity. They found reduced RNFL thickness and alterations in visual fields, visual evoked response (VER), and pattern ERG (pERG) in patients (see Box 4 for further details on the different types of ERG). Patients with greater damage in the RNFL tended to have lower quality of life and more severe symptoms. The RGC layer and the thickness of the peripapillary RNFL were inversely correlated with duration and severity of motor symptoms, and foveal thickness predicted the severity of the disease, alongside the overall decrease in quality of life. Electrophysiologically, decreased amplitudes of the pERG N95 component predicted lower quality of life scores. Following this, studying electrophysiological parameters, anatomical changes in the retina and global outcome of Parkinson’s disease has become increasingly more common. Structural and functional changes in the retina have been shown to correlate with disease duration and severity. With increasing duration and severity of the disease, increased pattern VER latency and decreased electrical activity on mfERG have been observed, potentially due to retinal thinning.^29,30^ The correlations between structural and functional changes and disease duration and severity reported by Kaur et al.^17^ suggest a retino-cortical structural–functional link supported by a connection between VER, contrast sensitivity and foveal thickness measures. The reduced foveal thickness and responses leading to VER deficits, as the occipital cortex is primarily and largely activated by macular neurons.^13,31^ However, several studies report no group difference in RNFL between Parkinson’s disease patients and control groups,^22,23,32–35^ even when electrophysiological changes such as longer latency and lower amplitude of the P100, lower contrast sensitivity, and worse visual field results are seen. This suggests that OCT may have lower sensitivity than electrophysiological assessments of retinal function in detecting changes associated with Parkinson’s disease,^36^ though it is also possible that the RNFL is less sensitive to disease progression than other layers. Another potential explanation is that neural dysfunction may begin several years before structural changes manifest from cell death or significant loss of dendrites or axons. Animal models of Parkinson’s disease are consistent with this, for example, showing swelling or dysmorphism of axons, before neuronal loss.^37,38^

### Relation to neuropathological postmortem retinal data

Besides in-vivo assessment of retinal changes, it is also possible to study changes in structure and function from post-mortem sampled retinas from Parkinson’s disease patients. Histological studies suggest that pathological α-synuclein accumulations are present in the retina, with lower retinal dopaminergic concentration in Parkinson’s disease patients at only 40% of the mean value for matched controls.^39,40^ Findings from immunocytochemical staining have revealed laminar alpha-synuclein inclusion in the INL; alpha-synuclein deposition in axons and dendrites within the IPL^41–43^ extracellular and intracellular alpha-synuclein inclusions in the RGCL and in dopaminergic amacrine cells at the border of the INL and IPL^41,42^ thinning of the inner layers, especially the INL; and both the central and the peripheral retina show alpha-synuclein pathology in Parkinson’s disease.^42^

α-synuclein has been found in all retinal layers and cells, predominantly in the retinal pigment epithelium, amacrine cells, and the IPL,^43^ and affected cells had aberrant structures such as (1) curly or swollen dendrites; (2) twisted structures; (3) intracytoplasmic accumulations of α-synuclein, and (4) axons not visibly emerging from any cell body with “beading” and swollen segments. Retinal α-synucleinopathy density scores positively correlate with brain α-synucleinopathy density scores, pathology stage and the UPDRS-III motor sub-score.

However, other studies did not find abnormal protein aggregation characteristic of Parkinson’s disease in the retinas of studied patients.^44^ In rodents, viral vector intravitreal injection showed a reduction in number of dopaminergic amacrine cells showing retinal alpha-synuclein accumulations in parallel to the central nervous system, and preceding development of clinical signs of disease.^45^

In Dementia with Lewy Bodies (DLB), a condition closely related to Parkinson’s disease (the main difference being the timing of onset of dementia),^46^ Devos et al.^47^ described abnormal enlargement of photoreceptors and intracellular pale inclusions in the outer plexiform layer in the retinal ganglion cell layer post-mortem. They suggest that these may relate to the increased latency and decreased amplitude of the photopic (light-adapted) and the scotopic (dark-adapted) electroretinography components seen in living patients with DLB.

Post-mortem studies and immunocytochemical staining evidence seem to be consistent with OCT findings, with thinning in most layers as well as OPL swelling being reported. Moreover, RNFL thinning, decreased macular volume and foveal thickness have also been shown in animal models using OCT scans alongside genetic analysis, translatable to visual and retinal irregularities in Parkinson’s disease patients.^41^

Beyond structural changes seen with OCT, the relationship between dopamine and the retina has also been examined. For example, reduced retinal dopamine was found post-mortem in patients who had not received dopaminergic treatment in life, compared with those on treatment.^39,48^ In animal models, reduced retinal dopamine is also linked with abnormal RGC pERG responses in monkeys.^49^ Therefore, electrophysiological impairments in Parkinson’s disease may relate to layer-specific anatomical abnormalities, including both thinning and swelling depending on the specific layer, which again can be linked to neurotransmitter and chemical deterioration as the disease progresses.

Finally, Huang et al.^13^ argued there was an important correlation between retinal morphology and function in Parkinson’s disease patients, and that a multi-parameter evaluation combining retinal structure and function can help differentiate patients with Parkinson’s disease and healthy controls. This stresses the importance of retinal morphology assessed by OCT because the origin of the decreased ERG activity can be attributed to anatomical and/or chemical etiologies. Retinal layer discrepancies or dopaminergic defects may thus explain abnormal ERG responses in Parkinson’s disease.

## Retinal electrophysiology

### Electroretinography in Parkinson’s disease

**Figure 2:**
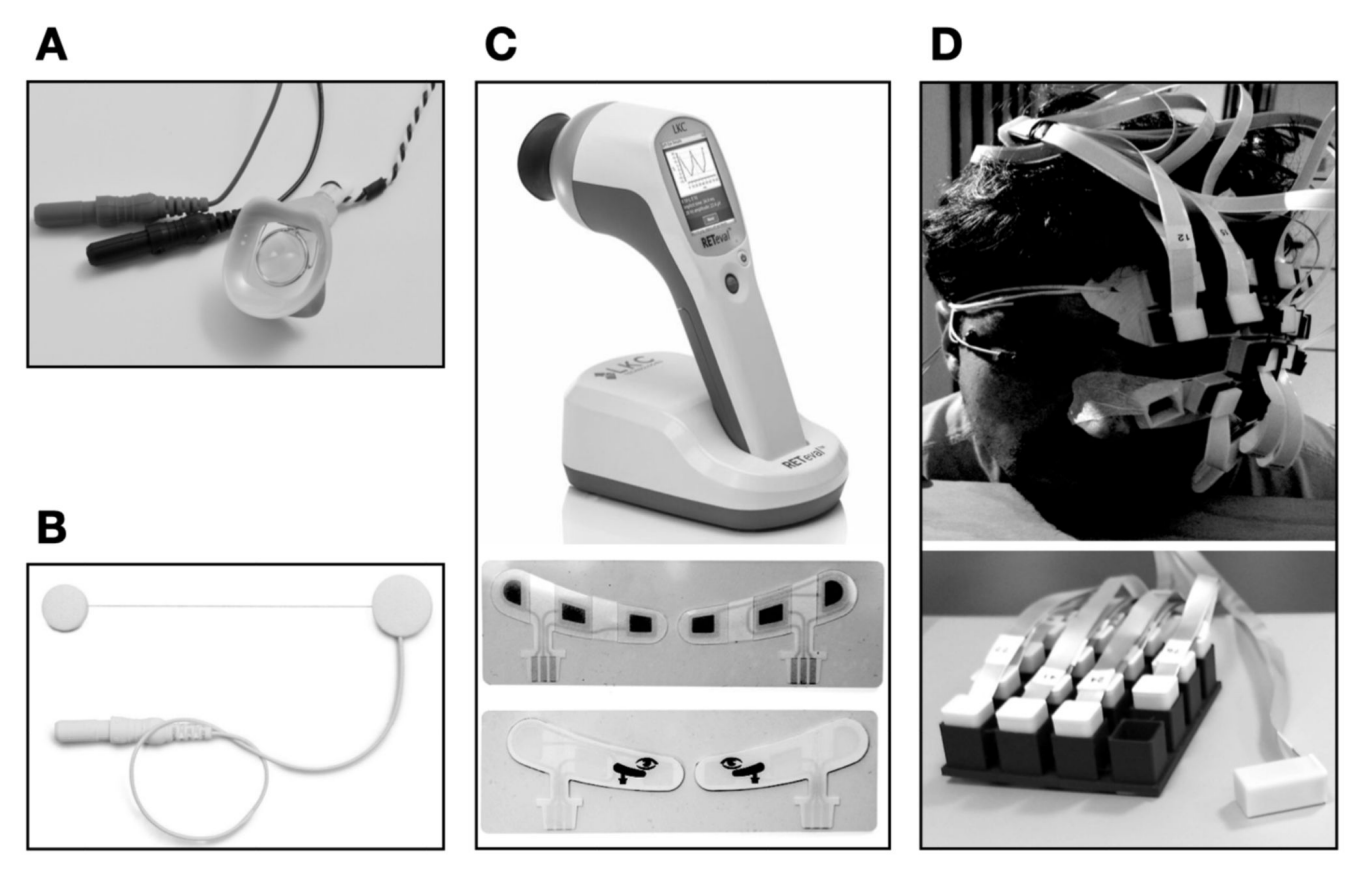
Different types of electroretinogram recording devices. (A) Burian-Allen corneal electrode. (B) Dawson-Trick-Litzkow electrode. (C) RETeval sensor strip electrodes. (D) Optically pumped magnetometers.

**Figure 3:**
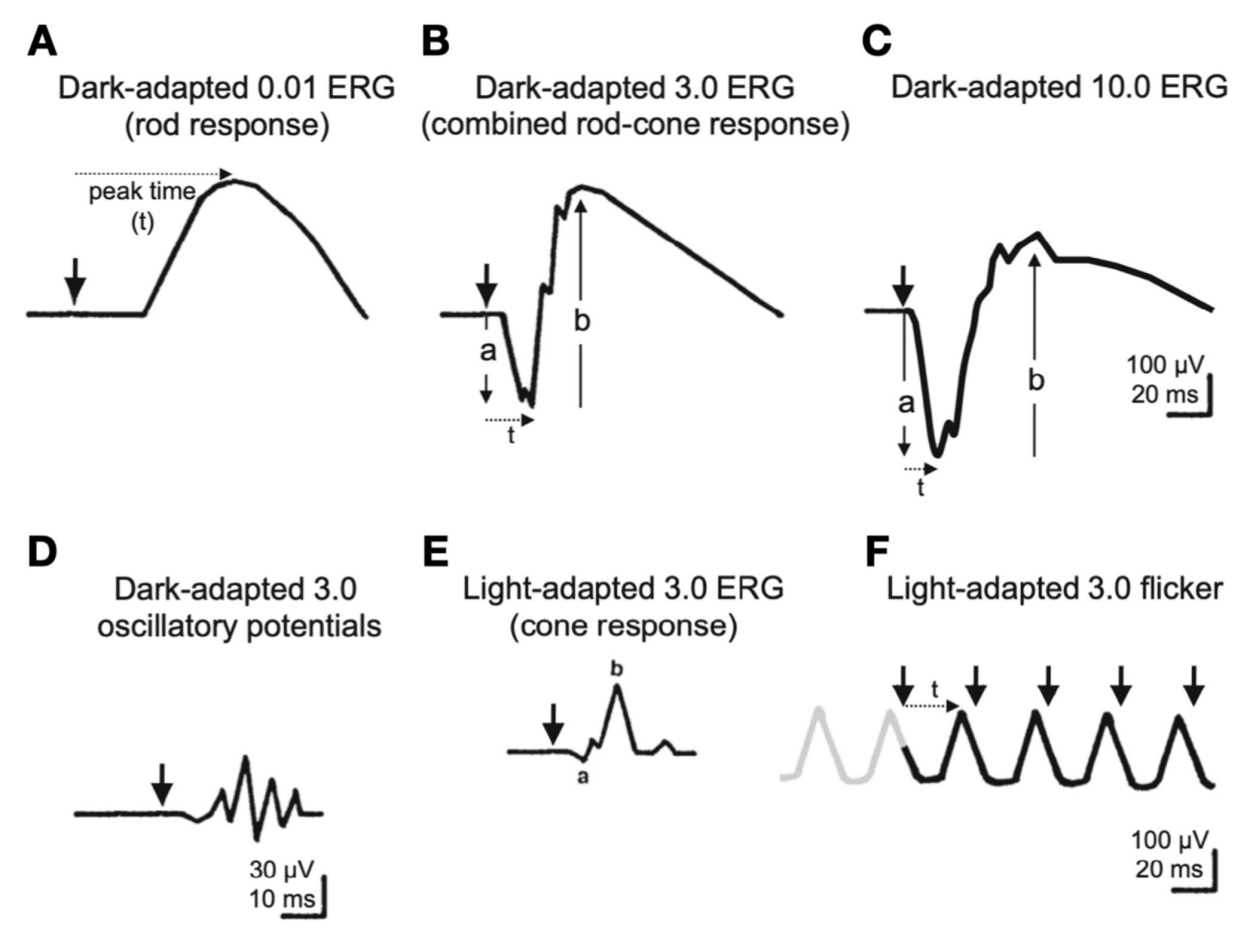
Diagram of basic electroretinograms defined by the ISCEV Standard from McCulloch *et al.*^71^ Reprinted with permission from [Springer Nature]: [Springer] [Documenta Ophthalmologica] [ISCEV Standard for full-field clinical electroretinography (2015 update), Daphne L. McCulloch et al), [License number: 5352540818755] (2015).

In the past decades, different ERG measures (see Box 2, Fig.2 for a description of ERG recording methods) have been shown to relate to Parkinson’s disease with more consistent and robust findings than those using OCT. Impaired retinal processing of stimulus contrast has been shown in patients with Parkinson’s disease (see Table 1), with significantly reduced amplitude in the ERG for low and medium spatial frequencies reported alongside reduced amplitude in ERG oscillatory potentials (see Box 3, Fig.3 for a description of classic ERG waveform, and Box 4, for description of ERG types).^56,57^ This decreased amplitude in electrophysiological components in ERG was associated to thinning of the retinal nerve fiber layer (RNFL), and the increased severity of overall retinal thinning measured with OCT was linked to decreased foveal electrical activity.^17,21,47^ Duration of disease and its severity were both correlated with electrophysiological changes in the retina and with increased latency in the cortical pattern visual evoked response in patients with Parkinson’s disease.^17^ Investigations of visual electrophysiology on patients with Parkinson’s disease revealed a prolonged P100 VER latency (113 ms in healthy controls vs. 126 ms in patients with Parkinson’s disease) which worsens as the disease progresses.^58^ A global decreased b-wave amplitude and increased latency of flash ERG (fERG) components^59–62^ and pERG spatial tuning^63,64^ are also seen. These become especially evident at later stages of disease.^60,65^ Recently, motor symptoms were linked to attenuated fERG amplitudes and shorter latencies.^59^ ERG has also been used with a rapid flicker stimulus to show that amplitude of cone-pathway responses are reduced at all flickering rates as the disease progresses.^60,66^

Similarly, electrical currents elicited by pERG from proximal retinal layers, such as ganglion cells, show lower amplitudes and a delay in the P50 component’s latency.^60,65,67^ Additionally, the pERG spatial frequency tuning peak (i.e., influence of pattern element size on the pERG amplitudes) is shifted towards higher frequencies, and amplitudes are lower when compared to matched controls (higher pERG amplitudes for stimuli with higher spatial frequencies).^63,64^ pERG can also be used to assess color function in the retina (chromatic pERG) and investigate potential dysfunction of the color pathways.^68^ Patients with Parkinson’s disease show different amplitude and peak latency for chromatic pERG for all colored stimuli with a distinct delay for blue and yellow stimuli.^68^ In animal studies, neurotoxin injections are linked with diminished retinal dopamine in rats, and abnormal pERG responses of retinal ganglion cells in monkeys with decreased numbers of amacrine cells. In turn, this leads to abnormal VER and ERG responses and decreased visual acuity and contrast sensitivity.^69^

Early investigations of the oscillatory potential failed to show any differences in Parkinson’s disease patients relative to healthy controls,^58^ but there have since been reports of an amplitude reduction of the overall index of the oscillatory potential and a prolongation of mean peak times.^29,35,49,60^ The discrepancies found in these later studies, when compared to earlier studies could be attributed to bigger sample size, more efficient patient group sampling, and improved recording techniques. mfERG to monitor functional changes in the macular and para-macular zone has revealed global reduced macular activity by decreased amplitudes in the mfERG of Parkinson’s disease patients.^13,14,21^ More specifically, these studies found latency delays in most of the mfERG rings but especially in the para-macular rings, alongside a reduced response amplitude in both macular and para-macular regions. Interestingly, both Moschos et al.21 and Huang et al.^13^ found strong internal consistency between retinal structure and retinal function even in patients recruited at early disease stages.

From these studies, it is clear that retinal electrophysiological dysfunctions are often seen in patients with Parkinson’s disease and are more consistently found than OCT changes. In general, there is a reduced amplitude and delayed response in Parkinson’s disease patients’ ERG in reaction to flashes, chromatic or achromatic checkerboard patterns that can be linked to the experienced visual symptoms. These effects can be seen at early stages and may relate more strongly to clinically relevant disease measures of severity.

## Dopaminergic influences on the electroretinogram

### Dopamine in the retina

Dopaminergic retinal cell loss has been linked to some of the visual symptoms in PD patients through the observation that they can be temporarily improved with administration of L-dopa. Dopamine is a key neurotransmitter in the retina, as well as in the brain. In the retina, it is synthesized by a subgroup of amacrine cells in the IPL, the axons of which reach the GCL and some also reach the OPL.^69,80^ Early descriptions showed similarities in the ERG, VER and contrast sensitivity between Parkinson’s disease patients and animal models with destroyed dopaminergic retinal cells81 and revealed an effect of dopamine antagonist injection on pERG tuning curves in primates.^82^ Dopamine may have a role in light adaptation in the retina due to its influence on photoreceptor transduction at the pre- and post-synaptic level.^83^ In rabbits, dopamine injections have been shown to influence retinal adaptation to light.^84^ Dopamine interacts with retinal circuitry at the origin of the field potential to suppress signal flow through rod circuits and enhance it through cone circuits.^40,85^

In previous experimental work in patient groups, dopamine and L-dopa have been shown to influence ERG and VER as well as oscillatory potentials. For example, an increase in b-wave amplitude and a decrease implicit time in scotopic conditions after administration of L-dopa has been recorded,^86^ demonstrating an overall effect of dopamine on retinal activity and an involvement of dopaminergic amacrine cells in retinal electrophysiology.^74,87^

### Effect of dopaminergic treatment on the ERG and on vision

Dopamine treatment has been shown to affect ERG measures. Even in very early studies, the longest P100 VER latencies were found in untreated patients,^58^ and decreased amplitude and increased fERG latencies as well as pERG tuning all improved with L-dopa treatment.^40,60,62,88^ Moreover, in healthy controls, the D2 antagonist l-sulpiride produces pERG alterations at medium spatial frequencies in the retina that are similar to those observed in Parkinson’s disease patients.^88^

Apart from electrophysiological abnormalities, dopamine also influences global aspects of visual perception such as contrast sensitivity, color vision, and visual acuity.^45,69,89^ Indeed, degeneration of dopaminergic amacrine cells and ganglion D cells are both associated with altered light-adaptation, reduced visual acuity, and impaired color discrimination. Consistent with this, L-dopa administration has been found to improve contrast sensitivity as well as chromatic pERG responses in patients with Parkinson’s disease.^69,89^

Degeneration of dopaminergic neurons in the retina may partly explain the retinal thinning observed in de novo Parkinson’s disease patients.^90^ This phenomenon was mostly observed in IPL and GCL and was linked to dopaminergic loss in the left substantia nigra, to disease severity, and to the Hoehn Yahr stage; reinforcing the possibility of a pathologic interconnection between the retina and nigral dopaminergic cell apoptosis. In addition, neurodegeneration of dopaminergic amacrine cells has been linked with alpha-synuclein overexpression in the eyes of patients with Parkinson’s disease and precedes the development of clinical signs.45 Local cytoplasmic inclusions and dopaminergic loss confirm the presence of neurodegenerative changes in the retina and potentially reflect similar changes in the central nervous system.^91^

Finally, other neurotransmitters beyond dopamine are involved in retinal processing. Disruptions of the pathways of neurotransmission inhibitors such as gamma-aminobutyric acid (GABA) will reduce or abolish oscillations in animal retina.^74^ Since dopaminergic amacrine cells send ascending signals to GABAergic synapses in both the INL and IPL cells,^92^ Parkinson’s disease may also affect GABAergic pathways. GABA is involved in the organization of ganglion and bipolar cells’ receptive fields and in the activity of photoreceptors. Dopamine also influences other retinal neurotransmitters such as excitatory glutamatergic and inhibitory glycinergic pathways.^40^

### Retinal measures and their role in Parkinson’s disease

Visual changes in patients with Parkinson’s disease have now been shown to relate to poorer outcomes such as higher rates of cognitive change^6,93,94^ as well as depression and even death.^5,95^ Color vision deficits, in particular, have been related to future cognitive decline and can even be found at early and prodromal stages.^96^ Some of these visual changes may reflect cortical processing, but some changes, especially color vision, and visual acuity and contrast sensitivity, are likely to relate to changes within the retina.^7,29,48,97^ The relative involvement of cortical and retinal processes can be disambiguated using techniques such as evoked responses. These can be obtained by recording electrical potentials from the scalp over the visual cortex.^98,99^ When used in combination with cortical measures, these can disentangle whether observed visual deficits arise from the retina, along the visual pathway or cerebral cortex. A recent study investigating the characteristics of the VER in patients with Parkinson’s disease found increased latencies in the cortical components with no cortical conduction time delays; supporting the view that this phenomenon may originate in the retina or along the pathway.^100,101^ Furthermore, besides a conduction delay, several VER component latencies were increased and related to severity of other clinically-relevant measures, including UPDRS motor scores and tests for visual selective attention.^100^ These increased latencies were even more strongly related in patients with Lewy body dementia^102^ and were linked to the presence of visual hallucinations.^103^

Although it is now well established that visual deficits in Parkinson’s disease patients relate to poorer outcomes, it is not yet clear whether these relate entirely to visual cortical dysfunction, or whether retinal changes are also predictive of Parkinson’s dementia. Longitudinal studies of retinal structure and function are lacking. One longitudinal study using OCT in Parkinson’s disease has shown a relationship between reduced retinal thickness and poorer cognition after three years104 but further studies are needed to determine the strength of this effect.

Finally, visual deficits can even be found in the prodromal stages of Parkinson’s disease, as early as decades before the onset of motor symptoms.^49,65,105^ Detection of visual changes at these premotor stages could potentially be of use in the future when disease-modifying therapies targeted at the earliest disease stages emerge. Taken together, these studies reinforce the potential role of the visual system in stratifying patients for differences in future outcomes. More specifically, there is a link between structural changes in the retina of patients with Parkinson’s disease, the electroretinographic and electrophysiological signatures of the impaired visual system, and the cognitive processing of downstream signal. This points to a potential role of retinal abnormalities as an additional marker of disease, and the benefit of routine retinal and visual assessment to help monitor disease progression at all stages of disease, and even in prodromal phases.

## Conclusion and future perspectives

Decreased visual acuity, abnormal spatial contrast sensitivity, difficulty in complex visual task completion, visual field defects, visual processing speeds disturbance, face and emotion recognition, and recurrent visual hallucinations are all visual impairments described in Parkinson’s disease. As these are becoming increasingly recognized, they are also being linked with poorer outcomes in Parkinson’s disease^6^, and efforts have been made in order to understand the origin of these symptoms. Although the optic tracts, LGN, and visual cortices are well-examined in previous studies, the importance of the retina may have been overlooked.^106^

Therefore, this work particularly focused on retinal changes in Parkinson’s disease: electrophysiology recorded through ERG, structural changes assessed using imaging techniques such as OCT and neurotransmission explored by means of postmortem retinal staining and animal models (for an overview, see Table 1).

Indicators such as increased retinocortical time, the signal propagation time between retina and cortex (measured with ERG and magnetoencephalography or electroencephalography) and changes in structure (imaged with OCT) could be valuable methods to detect and measure retinal changes in Parkinson’s disease. Electrophysiological assessment of retinal function in Parkinson’s disease has proven efficient in highlighting globally decreased response amplitude and conduction delay even at early stages of disease in a relatively non-invasive way. These techniques are accessible, cost effective, quick and have the potential to reveal useful insights to help stratify patients for more targeted treatments. Whether this has potential to help differentiate idiopathic Parkinson’s disease from atypical forms such as progressive supranuclear palsy or multiple systems atrophy remains to be determined.

## Figures and Tables

**Table 1:** Most reported retinal changes in Parkinson’s disease

		Effect reported	References
**Optical coherence tomography**	Macular retinal thickness	Decrease	13, 17, 20, 22, 24, 25
	Macular volume		13
	Retinal Nerve Fiber Layer		13, 14, 17-20, 86
	Retinal Ganglion Cell Layer	Decrease	14, 16, 86
		Increase	41, 42, 47
	Inner Plexiform Layer	Decrease	14, 16, 20, 86
	Inner Nuclear Layer		14, 16, 24, 42
	Outer Plexiform Layer	Decrease	14
		Increase	16, 47, 86
	Outer Nuclear Layer	Decrease	14, 16
	Pigment Epithelium		14
	Photoreceptor Layer	Decrease	14
		Increase	47
**Electroretinography**	Electroretinogram amplitudes	Decrease	13, 14, 21, 47, 60-66
	Oscillatory Potentials amplitudes		12^◊^, 35, 49, 60
	Oscillatory Potentials index		12^◊^, 35, 49, 60
	Electroretinogram latencies	Increase	13, 21, 47, 60, 66
**Retinal biochemistry**	Electroretinogram implicit time after L-Dopa	Decrease	40, 60, 86-88
	alpha-synuclein overexpression in retinal layers	Increase	39-43, 45
	Visual perception after L-Dopa	Improvement	45, 67, 89^◊^
	Electroretinogram amplitude after L-Dopa		40, 60, 86-88

◊ Citations are review articles.

ERG, electroretinogram.

## Box 1 Retinal anatomy

The retina is composed of distinct interconnected layers of neurons: three layers of nerve cell bodies and two layers of synapses (Figure 1) and is divided into central and peripheral regions.^9^ The first layer, the outer nuclear layer (ONL), contains cell bodies of the rods and cones and is about the same thickness in the central and peripheral retina. The second, the inner nuclear layer, contains bipolar, horizontal and amacrine cells, and a peripheral layer composed of cell bodies of amacrine cells. Finally, the innermost layer is the ganglion cell layers. Interspersed between these are synaptic layers where: (1) rods, cones, bipolar cells, and horizontal cells of the outer plexiform layer (OPL) connect, and where (2) the bipolar and the ganglion cells in the inner plexiform layer (IPL) are transmitting information to the ganglion cell layers, and ultimately to the brain along the optic nerve.^9,10^ Functionally, the photoreceptor layer transforms input light into electrical currents, while the ganglion cells send information to the visual cortex through the optic nerve. At the center of the retina, the concentration of photoreceptor cells increases, making it the region of the eye with the highest acuity. The central retina close to the fovea is considerably thicker than the peripheral retina.

*IPL, inner plexiform layer; ONL, outer nuclear layer; OPL, outer plexiform layer.* 

## Box 2 Methods for electroretinograms recording

The human electroretinogram (ERG) can be measured with several different types of specialized electrodes, such as the Burian-Allen contact lens corneal electrode (Fig. 2A); the Dawson-Trick-Litzkow (DTL) electrode (Fig. 2B) and the RETeval sensor strip electrode (Fig. 2C). Used in clinical and research settings for many years, the Burian-Allen electrode requires anesthesia and may result in an occasional corneal or conjunctival abrasion in users.^50^ The DTL electrode, a 2 cm long silver-impregnated nylon thread, is more comfortable to wear^51^ but electrode position can change during recording due to eye movements, affecting the measured amplitudes. The RETeval sensor strip electrodes (LKC Technologies, Inc., Gaithersburg, MD; Welch Allyn, Inc. Skaneateles Falls, NY) simultaneously measure full-field ERG and pupillary responses to light.^52^ The RETeval device is used alongside sensor strip electrodes that are less influenced by eye movements^53^ due to their placement on the lower lid but therefore also record lower amplitudes and have lower SNR than DTL electrodes.^54^ Recently, optically pumped magnetometers (Fig. 2D) have been shown to measure corresponding magnetic activity from the retina (magnetoretinogram) without any eye or skin contact at all, showing similar results to ERG recordings with DTL electrodes.^55^

*DTL, Dawson-Trick-Litzkow (electrode); ERG, electroretinogram* 

## Box 3 Electroretinography

The electroretinogram (ERG) describes the electrical response of the retina to visual stimuli.^70^ It can be evoked by flashes of light (full-field ERG), high-contrast checkerboard reversal (pattern ERG - pERG) and pseudorandom reversals of hexagonal patches (multifocal ERG - mfERG). There are different ways to record an ERG (cf. Box 2, Fig. 2) and standard guidelines for clinical electroretinography assessment are defined by the International Society for Clinical Electrophysiology of Vision (ISCEV).^71^ Using different flash intensity, measured in candela-seconds per meter squared (cd⋅s⋅m-2), it is possible to assess normal and pathological^72^ retinal function through electrophysiological activation: (Fig. 3A) dark-adapted b-wave rod-specific activity from ON-bipolar cells in the inner nuclear layer; (Fig. 3B) dark-adapted mixed rod-cones activation shown by the a-waves and b-waves where the a-wave (~15 ms) represents photoreceptor hyper-polarization; (Fig. 3C) dark-adapted test to differentiate photoreceptor and inner retina dysfunction; (Fig. 3D) oscillatory potentials arising from the amacrine cells; (Fig. 3E) light-adapted a-wave from cone photoreceptors and OFF-bipolar cells alongside light-adapted b-wave from ON-OFF bipolar cells; (Fig. 3F) light-adapted 30 Hz flicker to measure cone function.

*ERG, electroretinogram; mfERG, multifocal ERG; pERG, pattern ERG.* 

## Box 4 The different kinds of ERG

The flash ERG^59^ (fERG) is a full-field light flash stimulation indicative of cone or rod function in photopic and scotopic conditions. The waveform (Figure 2A-F) can be altered by a variety of retinal disorders, it is used to monitor disease progression^79^ and its mean amplitude decreases with age.^77^ Its main limitation stems from the summed activation from the whole retina which can lead to e.g., normal full-field fERG results for blind patients with macular degeneration.^73^

The multifocal ERG (mfERG) allows for investigation of ERG activity in small patches of retina.^78^ This technique measures local responses in an array of hexagonal elements in reaction to a pseudo-random high-rate sequence of illuminations. Each local ERG signal is mathematically extracted by correlating the continuous ERG signal with the sequence and the classic waveform is described as a negative deflection (N1) followed by a positive peak (P1) and another negative deflection (N2), different from the classic a- and b-wave features.^75^ The mfERG can show attenuated ERGs in case of retinal pathology. The pattern ERG (pERG) evokes responses using a contrast-reversing black and white checkerboard pattern.^76^ The pERG elicits three components, a negative deflection around 35 ms (N35), a positive P50 representing macular photoreceptors activity and a negative N95 arising from the ganglion cell layer.^72,75^ It is mostly a transient response that can be used in order to assess the integrity of the photoreceptors, and the bipolar and ganglion cells via amplitude reductions or peak delays. The oscillatory potentials can be observed after a fERG and are low-amplitude high-frequency ripples superimposed on the rising flank of the b-wave, identified by filtering. Their origin is still under debate and could stem from amacrine cells or ganglion cells in the inner retinal layers.^66,74^ Clinically, abnormal oscillatory potentials can indicate mild retinal ischemia in the inner retina and is used as an indicator of potential retinopathy.

*ERG, electroretinography; fERG, flash ERG; mfERG, multifocal ERG; pERG, pattern ERG.* 

## Abbreviations

DTL: Dawson-Trick-Litzkow (electrode)
ERG: electroretinography
fERG: flash ERG
GABA: gamma-Aminobutyric acid
INL: inner nuclear layer
IPL: inner plexiform layer
ISCEV: International Society for Clinical Electrophysiology of Vision
L-dopa: levodopa
mfERG: multifocal ERG
OCT: optical coherence tomography
ONL: outer nuclear layer
OPL: outer plexiform layer
pERG: pattern ERG
RGCIPL: retinal ganglion cells inner plexiform layer
RNFL: retinal nerve fiber layer
UPDRS: Unified Parkinson’s Disease Rating Scale
VER: visual evoked response

## References

[1] Urwyler P, Nef T, Killen A (2014). Visual complaints and visual hallucinations in Parkinson’s disease. Parkinsonism Relat Disord.

[2] Ekker MS, Janssen S, Seppi K (2017). Ocular and visual disorders in Parkinson’s disease: Common but frequently overlooked. Parkinsonism Relat Disord.

[3] Davidsdottir S, Cronin-Golomb A, Lee A (2005). Visual and spatial symptoms in Parkinson’s disease. Vision Res.

[4] Archibald NK, Clarke MP, Mosimann UP (2011). Visual symptoms in Parkinson’s disease and Parkinson’s disease dementia. Mov Disord.

[5] Hamedani AG, Abraham DS, Maguire MG (2020). Visual Impairment Is More Common in Parkinson’s Disease and Is a Risk Factor for Poor Health Outcomes. Mov Disord.

[6] Zarkali A, McColgan P, Leyland LA (2021). Visual Dysfunction Predicts Cognitive Impairment and White Matter Degeneration in Parkinson’s Disease. Mov Disord.

[7] Weil RS, Schrag AE, Warren JD (2016). Visual dysfunction in Parkinson’s disease. Brain.

[8] Leyland LA, Bremner FD, Mahmood R (2020). Visual tests predict dementia risk in Parkinson disease. Neurol Clin Pract.

[9] Kolb H, Fernandez E, Nelson R (2011). Webvision : the organization of the retina and visual system.

[10] Masland Richard H (2012). The Neuronal Organization of the Retina. Neuron.

[11] Jindahra P, Hedges TR, Mendoza-Santiesteban CE (2010). Optical coherence tomography of the retina: applications in neurology. Curr Opin Neurol.

[12] Cova I, Priori A (2018). Diagnostic biomarkers for Parkinson’s disease at a glance: where are we?. J Neural Transm (Vienna).

[13] Huang J, Li Y, Xiao J (2018). Combination of Multifocal Electroretinogram and Spectral-Domain OCT Can Increase Diagnostic Efficacy of Parkinson’s Disease. Parkinsons Dis.

[14] Unlu M, Gulmez Sevim D, Gultekin M (2018). Correlations among multifocal electroretinography and optical coherence tomography findings in patients with Parkinson’s disease. Neurol Sci.

[15] Hoehn MM, Yahr MD (1967). Parkinsonism: onset, progression and mortality. Neurology.

[16] Chorostecki J, Seraji-Bozorgzad N, Shah A (2015). Characterization of retinal architecture in Parkinson’s disease. J Neurol Sci.

[17] Kaur M, Saxena R, Singh D (2015). Correlation Between Structural and Functional Retinal Changes in Parkinson Disease. J Neuroophthalmol.

[18] Rohani M, Langroodi AS, Ghourchian S (2013). Retinal nerve changes in patients with tremor dominant and akinetic rigid Parkinson’s disease. Neurol Sci.

[19] Garcia-Martin E, Rodriguez-Mena D, Satue M (2014). Electrophysiology and optical coherence tomography to evaluate Parkinson disease severity. Invest Ophthalmol Vis Sci.

[20] Sari ES, Koc R, Yazici A (2015). Ganglion cell-inner plexiform layer thickness in patients with Parkinson disease and association with disease severity and duration. J Neuroophthalmol.

[21] Moschos MM, Tagaris G, Markopoulos L (2011). Morphologic Changes and Functional Retinal Impairment in Patients with Parkinson Disease without Visual Loss. Eur J Ophthalmol.

[22] Archibald NK, Clarke MP, Mosimann UP (2011). Retinal thickness in Parkinson’s disease. Parkinsonism Relat Disord.

[23] Tsironi EE, Dastiridou A, Katsanos A (2012). Perimetric and retinal nerve fiber layer findings in patients with Parkinson’s disease. BMC Ophthalmol.

[24] Lee JY, Kim JM, Ahn J (2014). Retinal nerve fiber layer thickness and visual hallucinations in Parkinson’s Disease. Mov Disord.

[25] Inzelberg R, Ramirez JA, Nisipeanu P (2004). Retinal nerve fiber layer thinning in Parkinson disease. Vision Res.

[26] Liu L, Jia Y, Takusagawa HL (2015). Optical Coherence Tomography Angiography of the Peripapillary Retina in Glaucoma. JAMA ophthalmology.

[27] Sung MS, Choi S-M, Kim J (2019). Inner retinal thinning as a biomarker for cognitive impairment in de novo Parkinson’s disease. Sci Rep.

[28] Murueta-Goyena A, del Pino R, Reyero P (2019). Parafoveal thinning of inner retina is associated with visual dysfunction in Lewy body diseases. Mov Disord.

[29] Gottlob I, Schneider E, Heider W (1987). Alteration of visual evoked potentials and electroretinograms in Parkinson’s disease. Electroencephalogr Clin Neurophysiol.

[30] Huang J, Beach P, Bozoki A (2020). Alzheimer’s Disease Progressively Alters the Face-Evoked Visual-Processing Network. Journal of Alzheimer’s Disease.

[31] Miri S, Glazman S, Mylin L (2016). A combination of retinal morphology and visual electrophysiology testing increases diagnostic yield in Parkinson’s disease. Parkinsonism Relat Disord.

[32] Aaker GD, Myung JS, Ehrlich JR (2010). Detection of retinal changes in Parkinson’s disease with spectral-domain optical coherence tomography. Clin Ophthalmol.

[33] Albrecht P, Müller A-K, Südmeyer M (2012). Optical Coherence Tomography in Parkinsonian Syndromes. PLoS One.

[34] Nowacka B, Lubinski W, Honczarenko K (2014). Ophthalmological features of Parkinson disease. Med Sci Monit.

[35] Nowacka B, Lubiński W, Honczarenko K (2015). Bioelectrical function and structural assessment of the retina in patients with early stages of Parkinson’s disease (PD). Doc Ophthalmol.

[36] Hasanov S, Demirkilinc Biler E, Acarer A (2019). Functional and morphological assessment of ocular structures and follow-up of patients with early-stage Parkinson’s disease. Int Ophthalmol.

[37] Chung CY, Koprich JB, Siddiqi H (2009). Dynamic Changes in Presynaptic and Axonal Transport Proteins Combined with Striatal Neuroinflammation Precede Dopaminergic Neuronal Loss in a Rat Model of AAV α-Synucleinopathy. The Journal of Neuroscience.

[38] Li Y, Liu W, Oo TF (2009). Mutant LRRK2(R1441G) BAC transgenic mice recapitulate cardinal features of Parkinson’s disease. Nat Neurosci.

[39] Harnois C, Di Paolo T (1990). Decreased dopamine in the retinas of patients with Parkinson’s disease. Invest Ophthalmol Vis Sci.

[40] Witkovsky P (2004). Dopamine and retinal function. Doc Ophthalmol.

[41] Beach TG, Carew J, Serrano G (2014). Phosphorylated α-synuclein-immunoreactive retinal neuronal elements in Parkinson’s disease subjects. Neurosci Lett.

[42] Bodis-Wollner I, Kozlowski PB, Glazman S (2014). α-synuclein in the inner retina in parkinson disease. Ann Neurol.

[43] Ortuño-Lizarán I, Beach TG, Serrano GE (2018). Phosphorylated α-synuclein in the retina is a biomarker of Parkinson’s disease pathology severity. Mov Disord.

[44] Ho C-Y, Troncoso JC, Knox D (2014). Beta-amyloid, phospho-tau and alpha-synuclein deposits similar to those in the brain are not identified in the eyes of Alzheimer’s and Parkinson’s disease patients. Brain Pathol.

[45] Marrocco E, Indrieri A, Esposito F (2020). α-synuclein overexpression in the retina leads to vision impairment and degeneration of dopaminergic amacrine cells. Sci Rep.

[46] McKeith IG, Boeve BF, Dickson DW (2017). Diagnosis and management of dementia with Lewy bodies. Neurology.

[47] Devos D, Tir M, Maurage CA (2005). ERG and anatomical abnormalities suggesting retinopathy in dementia with Lewy bodies. Neurology.

[48] Archibald NK, Clarke MP, Mosimann UP (2009). The retina in Parkinson’s disease. Brain.

[49] Veys L, Vandenabeele M, Ortuno-Lizaran I (2019). Retinal alpha-synuclein deposits in Parkinson’s disease patients and animal models. Acta Neuropathol.

[50] Dawson WW, Trick GL, Litzkow CA (1979). Improved electrode for electroretinography. Invest Ophthalmol Vis Sci.

[51] Al-Otaibi H, Al-Otaibi MD, Khandekar R (2017). Validity, Usefulness and Cost of RETeval System for Diabetic Retinopathy Screening. Transl Vis Sci Technol.

[52] Tang J, Hui F, Hadoux X (2018). A Comparison of the RETeval Sensor Strip and DTL Electrode for Recording the Photopic Negative Response. Transl Vis Sci Technol.

[53] Brouwer AH, de Wit GC, de Boer JH (2020). Effects of DTL electrode position on the amplitude and implicit time of the electroretinogram. Doc Ophthalmol.

[54] Man TTC, Yip YWY, Cheung FKF (2020). Evaluation of Electrical Performance and Properties of Electroretinography Electrodes. Transl Vis Sci Technol.

[55] Westner BU, Lubell JI, Jensen M, Hokland S, Dalal SS (2021). Contactless measurements of retinal activity using optically pumped magnetometers. Neuroimage.

[56] Calzetti S, Franchi A, Taratufolo G (1990). Simultaneous VEP and PERG investigations in early Parkinson’s disease. J Neurol Neurosurg Psychiatry.

[57] Langheinrich T, Tebartz van Elst L, Lagrèze WA (2000). Visual contrast response functions in Parkinson’s disease: evidence from electroretinograms, visually evoked potentials and psychophysics. Clin Neurophysiol.

[58] Kupersmith MJ, Shakin E, Siegel IM (1982). Visual System Abnormalities in Patients With Parkinson’s Disease. Arch Neurol.

[59] Netser R, Demmin DL, Dobkin R (2021). Flash Electroretinography Parameters and Parkinson’s Disease. J Parkinsons Dis.

[60] Ikeda H, Head GM, K Ellis CJ (1994). Electrophysiological signs of retinal dopamine deficiency in recently diagnosed Parkinson’s disease and a follow up study. Vision Res.

[61] Gelmi C, Sandrini G, Martignoni E (1992). Electroretinograms and visual evoked cortical potentials in Parkinsonian patients with or without L-Dopa treatment. NeuroOphthalmol.

[62] Brandies R, Yehuda S (2008). The possible role of retinal dopaminergic system in visual performance. Neurosci Biobehav Rev.

[63] Tagliati M, Bodis-Wollner I, Yahr MD (1996). The pattern electroretinogram in Parkinson’s disease reveals lack of retinal spatial tuning. Electroencephalography and Clinical Neurophysiology/Evoked Potentials Section.

[64] Peppe A, Stanzione P, Pierantozzi M (1998). Does pattern electroretinogram spatial tuning alteration in Parkinson’s disease depend on motor disturbances or retinal dopaminergic loss?. Electroencephalogr Clin Neurophysiol.

[65] Nightingale S, Mitchell KW, Howe JW (1986). Visual evoked cortical potentials and pattern electroretinograms in Parkinson’s disease and control subjects. J Neurol Neurosurg Psychiatry.

[66] Perlman I, Kolb H, Fernandez E, Nelson R (1995). Webvision: The Organization of the Retina and Visual System.

[67] Peppe A, Stanzione P, Pierelli F (1995). Visual alterations in de novo Parkinson’s disease: pattern electroretinogram latencies are more delayed and more reversible by levodopa than are visual evoked potentials. Neurology.

[68] Sartucci F, Orlandi G, Bonuccelli U (2006). Chromatic pattern-reversal electroretinograms (ChPERGs) are spared in multiple system atrophy compared with Parkinson’s disease. Neurol Sci.

[69] Indrieri A, Pizzarelli R, Franco B (2020). Dopamine, Alpha-Synuclein, and Mitochondrial Dysfunctions in Parkinsonian Eyes. Front Neurosci.

[70] Vincent A, Robson AG, Holder GE (2013). Pathognomonic (diagnostic) ERGs. A review and update. Retina.

[71] McCulloch DL, Marmor MF, Brigell MG (2015). ISCEV Standard for full-field clinical electroretinography (2015 update). Doc Ophthalmol.

[72] Tsang SH, Sharma T, Tsang SH, Sharma T (2018). Atlas of Inherited Retinal Diseases.

[73] Creel DJ, Levin KH, Chauvel P (2019). Handb Clin Neurol.

[74] Wachtmeister L (1998). Oscillatory potentials in the retina: what do they reveal. Prog Retin Eye Res.

[75] Bach M, Brigell MG, Hawlina M (2013). ISCEV standard for clinical pattern electroretinography (PERG): 2012 update. Doc Ophthalmol.

[76] Hood DC, Bach M, Brigell M (2012). ISCEV standard for clinical multifocal electroretinography (mfERG) (2011 edition). Doc Ophthalmol.

[77] Birch DG, Anderson JL (1992). Standardized Full-Field Electroretinography: Normal Values and Their Variation With Age. Arch Ophthalmol.

[78] Sutter EE, Dartt DA (2010). Encyclopedia of the Eye.

[79] Gauvin M, Dorfman AL, Lachapelle P, Boon CJF, Wijnholds J (2018). Retinal Gene Therapy: Methods and Protocols.

[80] Popova E (2014). Role of dopamine in distal retina. J Comp Physiol A Neuroethol Sens Neural Behav Physiol.

[81] Nguyen-Legros J (1988). Functional neuroarchitecture of the retina: hypothesis on the dysfunction of retinal dopaminergic circuitry in Parkinson’s disease. Surg Radiol Anat.

[82] Tagliati M, Bodis-Wollner I, Kovanecz I (1994). Spatial frequency tuning of the monkey pattern ERG depends on D2 receptor-linked action of dopamine. Vision Res.

[83] Jackson CR, Ruan G-X, Aseem F (2012). Retinal dopamine mediates multiple dimensions of light-adapted vision. J Neurosci.

[84] Gottvall E, Textorius O (2003). Concentration-dependent effects of dopamine on the direct current electroretinogram of pigmented rabbits during prolonged intermittent recording. Doc Ophthalmol.

[85] Bodis-Wollner I (1990). Visual deficits related to dopamine deficiency in experimental animals and Parkinson’s disease patients. Trends Neurosci.

[86] Silverstein SM, Demmin DL, Schallek JB (2020). Measures of Retinal Structure and Function as Biomarkers in Neurology and Psychiatry. Biomark Neuropsychiatry.

[87] Gottlob I, Weghaupt H, Vass C (1989). Effect of levodopa on the human pattern electroretinogram and pattern visual evoked potentials. Graefes Arch Clin Exp Ophthalmol.

[88] Stanzione P, Bodis-Wollner I, Pierantozzi M (1999). A mixed D1 and D2 antagonist does not replay pattern electroretinogram alterations observed with a selective D2 antagonist in normal humans: relationship with Parkinson’s disease pattern electroretinogram alterations. Clin Neurophysiol.

[89] Guo L, Normando EM, Shah PA (2018). Oculo-visual abnormalities in Parkinson’s disease: Possible value as biomarkers. Mov Disord.

[90] Ahn J, Lee J-Y, Kim TW (2018). Retinal thinning associates with nigral dopaminergic loss in de novo Parkinson disease. Neurology.

[91] Kashani AH, Asanad S, Chan JW (2021). Past, present and future role of retinal imaging in neurodegenerative disease. Prog Retin Eye Res.

[92] Armstrong RA (2015). Oculo-Visual Dysfunction in Parkinson’s Disease. J Parkinsons Dis.

[93] Williams-Gray CH, Mason SL, Evans JR (2013). The CamPaIGN study of Parkinson's disease: 10-year outlook in an incident population-based cohort. Journal of Neurology, Neurosurgery &amp; Psychiatry.

[94] Anang JB, Gagnon JF, Bertrand JA (2014). Predictors of dementia in Parkinson disease: a prospective cohort study. Neurology.

[95] Han G, Han J, Han K (2020). Visual Acuity and Development of Parkinson’s Disease: A Nationwide Cohort Study. Mov Disord.

[96] Fereshtehnejad S-M, Yao C, Pelletier A (2019). Evolution of prodromal Parkinson’s disease and dementia with Lewy bodies: a prospective study. Brain.

[97] Armstrong RA (2017). Visual Dysfunction in Parkinson’s Disease. Int Rev Neurobiol.

[98] Bodis-Wollner I, Yahr MD (1978). Measurements of Visual Evoked Potentials in Parkinson’s Disease. Brain.

[99] Creel DJ, Levin KH, Chauvel P (2019). Handb Clin Neurol.

[100] Naganuma R, Yabe I, Takeuchi M (2020). Clinical factors affecting evoked magnetic fields in patients with Parkinson’s disease. PLoS One.

[101] Fujisawa Y, Minato T, Uemura JI (2017). Association between changes in visual evoked magnetic fields and non-motor features in Parkinson’s disease. Nagoya J Med Sci.

[102] Carrarini C, Russo M, Pagliaccio G (2021). Visual evoked potential abnormalities in dementia with Lewy bodies. Neurophysiol Clin.

[103] Murphy N, Killen A, Gupta RK (2021). Exploring Bottom-Up Visual Processing and Visual Hallucinations in Parkinson’s Disease With Dementia. Front Neurol.

[104] Murueta-Goyena A, Del Pino R, Galdós M (2021). Retinal Thickness Predicts the Risk of Cognitive Decline in Parkinson Disease. Ann Neurol.

[105] Mahlknecht P, Seppi K, Poewe W (2015). The Concept of Prodromal Parkinson’s Disease. J Parkinsons Dis.

[106] Prasad S, Galetta SL, Kennard C, Leigh RJ (2011). Handb Clin Neurol.

